# Oxygen therapy in palliative care

**DOI:** 10.1038/npjpcrm.2015.73

**Published:** 2016-01-07

**Authors:** H John Fardy

**Affiliations:** 1 Graduate School of Medicine, University of Wollongong, Wollongong, NSW, Australia

## Abstract

Breathlessness in advanced disease is a common problem, with the majority of people experiencing breathlessness in the weeks before death. The thrust of the new British Thoracic Society guidelines for home oxygen in adults is that oxygen therapy for home use is most useful in chronic hypoxaemia. However, clinicians make individual clinical decisions, cognisant of the guidelines but ultimately determined by what relieves the symptoms of the individual most effectively.

The recent publication of the article ‘When should I be considering home oxygen for my patients’ is timely.^1^ On the basis of the new British Thoracic Society guidelines for home oxygen in adults,^2^ it is a practical summary and the explanation of when to use oxygen therapy by means of developing case studies is helpful.

Oxygen is a therapy and, as a result, has benefits, but also risks. We are all aware of the ‘carbon dioxide retainer’ in chronic obstructive pulmonary disease (COPD), but there are also other situations in which oxygen therapy has risks. It is a drug that must be dosed and monitored appropriately.

The thrust of the guideline is that oxygen therapy for home use is most useful in chronic hypoxaemia. If the person is breathless, but not hypoxic, oxygen is generally not indicated. Alternative ways of dealing with breathlessness include the moving of air across the dermatomes of the trigeminal nerve (‘fan therapy’). Oral morphine in low doses (the dose is much less than for pain) is the drug of choice for breathlessness.

Breathlessness in advanced disease is a common problem (see Table 1),^3^ with 65% of people experiencing breathlessness in the weeks before death.^4^ Particularly in the palliative care setting, many patients have multimorbidity and sometimes the breathlessness is unrelated to their palliative care problem. A patient who has a malignancy may also have heart failure, anaemia, recent-onset pleural effusion, hypothyroid disease or undiagnosed and therefore untreated COPD. All these diagnoses can present as breathlessness. In addition, having dyspnoea is anxiety-provoking and being anxious can provoke dyspnoea. The strategy of (i) adequate history, (ii) appropriate examination, (iii) consideration of this patient’s problem and finally (iv) consideration of special investigations will hopefully lead to a diagnosis.

Guidelines make absolute recommendations. If a patient with advanced disease is breathless and hypoxic, oxygen is the preferred treatment; if they are breathless and not hypoxic, oxygen is not recommended. However, clinicians will (and should) make individual clinical decisions, cognisant of the guidelines but ultimately determined by what relieves the symptoms of the individual most effectively. The art of medicine lies in deciding, with the patient, what is reasonable treatment for this patient at this time.

## Figures and Tables

**Table 1:** Prevalence of breathlessness in advanced disease^3^

	*Estimated prevalence of breathlessness in advanced disease*
Cancer	10–70%
AIDS	11–62%
Heart disease	60–88%
Chronic obstructive pulmonary disease	90–95%
Chronic kidney disease	11–62%
